# Acute effects of high-intensity interval training on adiponectin isoforms in inactive young adults: a quasi-experimental study

**DOI:** 10.1186/s13102-025-01433-7

**Published:** 2025-12-30

**Authors:** Daniela Alvarado-Carrasco, Antonio González-Maraboli, María Reyes-Montalva, Josefa Videla-Aguilar, Amanda Bentes, Luis Peñailillo, Viviana Sandoval, Sergio Martínez-Huenchullán

**Affiliations:** 1 Escuela de Kinesiología, Facultad de Medicina, Universidad Austral de Chile, Valdivia, Chile; 2 Instituto de Anatomía, Patología e Histología, Facultad de Medicina, Universidad Austral de Chile, Valdivia, Chile; 3 Exercise and Rehabilitation Sciences Institute, School of Physical Therapy, Faculty of Rehabilitation Sciences, Universidad Andres Bello, Santiago, Chile; 4 Carrera de Nutrición y Dietética, Facultad de Ciencias de la Rehabilitación y Calidad de Vida, Universidad San Sebastián, Valdivia, Chile; 5 Carrera de Kinesiología, Facultad de Ciencias de la Rehabilitación y Calidad de Vida, Universidad San Sebastián, Valdivia, Chile; 6 Centro de Biología Celular y Biomedicina (CEBICEM), Facultad de Ciencias, Universidad San Sebastián, Santiago, Chile

**Keywords:** Adiponectin, High-intensity interval training, Sedentary behavior

## Abstract

**Background:**

Physical inactivity is a key risk factor for metabolic disorders. High-intensity interval training (HIIT) has been recognized for its metabolic benefits, but its acute effects on adiponectin isoforms remain unclear. This study examines the impact of a single HIIT session on circulating adiponectin (high-molecular weight; HMW, medium-molecular-weight; MMW, and low-molecular weight; LMW) isoforms and metabolic outcomes in insufficiently active young adults.

**Methods:**

A quasi-experimental study was conducted with 21 physically inactive adults (11 women, 10 men, mean age 22.7 ± 1.5 years). Participants performed a HIIT session consisting of six bouts at 80% heart rate reserve (HRR). Pre- and post-exercise blood samples were ana-lyzed for adiponectin isoforms via Western blotting, and metabolic markers were assessed. Statistical analyses included Wilcoxon tests.

**Results:**

MMW adiponectin significantly increased (~ 19%; effect size [95%CI]: 0.49 [-0.02-1.00] *p* < 0.05), while LMW and HMW adiponectin remained unchanged. The HMW/MMW ratio decreased (-16%; effect size [95%CI]: -0.49 [-0.99-0.00] *p* < 0.05). Additionally, non-HDL cholesterol (effect size [95%CI]: 0.464 [-0.03-0.958]) and lactate levels (effect size [95%CI]: 1.164 [0.568–1.759]) increased post-exercise (both *p* < 0.05).

**Conclusions:**

A single HIIT session acutely increases MMW adiponectin in insufficiently active young adults. Further research is needed to determine the long-term metabolic implications of repeated HIIT sessions on adiponectin isoform dynamics.

**Trial registration:**

retrospectively registered in ClinicalTrials.gov (NCT07146867) on August 21st, 2025.

**Supplementary Information:**

The online version contains supplementary material available at 10.1186/s13102-025-01433-7.

## Background

Physical inactivity, defined as non-compliance with general recommendations for weekly physical activity levels [1], is a major risk factor for cardiometabolic diseases worldwide [2]. Its growing prevalence among young adults [3] contributes to an increased incidence of hypertension, dyslipidemia, insulin resistance, and type 2 diabetes [4]. Physical exercise remains one of the most effective non-pharmacological strategies to counter these risks [5, 6].

Among exercise modalities, high-intensity interval training (HIIT) has emerged as an efficient approach to improving metabolic health in physically inactive individuals [7, 8], achieving benefits comparable to moderate-intensity continuous training (MICT) [9] despite shorter exercise durations [10, 11]. Chronic HIIT interventions (≥ 2 sessions per week for ≥ 2 weeks) have been shown to enhance glucose and lipid metabolism, particularly in individuals with type 2 diabetes [12] and in adults with overweight or obesity [13]. In addition, acute HIIT sessions can lower postprandial glucose for up to 24 h in adults with obesity [14, 15] and reduce the expression of toll-like receptors (TLR) 2 and 4 in lymphocytes and monocytes after short-term training [16], suggesting favorable metabolic and inflammatory adaptations.

Adiponectin and its molecular weight isoforms have been identified as key mediators of the metabolic effects of exercise [17], particularly HIIT [18]. This ~ 30 kDa protein functions as an anti-inflammatory, insulin sensitizer, and β-oxidation stimulant and exists in different molecular weight isoforms that form distinct complexes [19]. The most commonly described adiponectin isoforms include low-molecular-weight (LMW; trimer), medium-molecular-weight (MMW; hexamer), and high-molecular-weight (HMW) adiponectin. Among them, the higher molecular weight complexes exhibit greater bioactivity [20], with the HMW isoform consisting of multimers of 12 or more adiponectin monomers. Elevated levels of HMW adiponectin have been associated with a lower risk of developing peripheral artery disease [21], whereas lower HMW-to-total adiponectin ratios have been linked to an increased risk of cardiovascular alterations, such as coronary plaque formation [22]. Interestingly, plasma adiponectin levels are associated with lipid profile parameters, such as triglycerides, low, and very-low density lipoproteins [23], and lactate [24]. Therefore, they have been raised as potential factors involved in adiponectin metabolism.

Regarding the effects of exercise, the adiponectin response appears to be intensity-dependent. Recently, Mallardo et al. reported significant increases in both total and HMW adiponectin in young amateur male athletes after a single session of exhaustive exercise [25]. In individuals with obesity, similar increases in total adiponectin have been observed in women following a single session of HIIT, although only total adiponectin levels were reported in that study [26]. Furthermore, a previous study from our group found no significant changes in circulating adiponectin isoforms after a moderate-intensity continuous training (MICT) session in physically inactive young adults [27]. Based on these findings, we hypothesized that the adiponectin isoform response may be dependent on exercise intensity.

Building on this evidence, the aim of this report was to evaluate the acute metabolic effects of one HIIT session in physically inactive young adults, with a particular focus on adiponectin isoforms and, secondarily, on glucose and lipid metabolism outcomes. We hypothesize that a single HIIT session will increase circulating adiponectin, specifically the higher molecular weight isoforms (MMW and HMW), which are known to have greater metabolic bioactivity.

## Methods

### Study design

This study was reviewed and approved by the Scientific Ethics Committee of the Los Ríos Health Service (code 443/2023) and was retrospectively registered in ClinicalTrials.gov (NCT07146867) on August 21 st, 2025. Prior to participation, volunteers signed an informed consent form, agreeing to their voluntary involvement. This is a quasi-experimental study. For the sample size calculation, an effect size of 0.76 was considered (based on changes in plasma insulin after a single exercise session seen by our group in a previous study) [27], with a 5% of error probability (α), 95% of statistical power (β), and a pre- and post-exercise correlation of 0.6. As a result, the expected sample size for this study was 20 participants. Changes in circulating insulin were used to estimate the effect size, considering the close association between alterations in adiponectin levels and insulin resistance in humans [28].

### Participants

The recruitment of participants was conducted through convenience sampling using social media, posters, and word-of-mouth. The inclusion criteria were as follows: age between 18 and 29 years, engage in less than 150 min of moderate-intensity or 75 min of vigorous-intensity physical activity per week), and having no positive responses on the PAR-Q & YOU questionnaire [29]. Exclusion criteria included a diagnosis of cardiometabolic disease or the use of anti-inflammatory treatment during the week prior to participation. Both men and women were included in the study to allow for broader extrapolation of the findings to the general population, in line with current recommendations when sex is not a specific research variable [30], as shown in Table 1.

**Table 1:** Characteristics of the participants

Parameter	Female (*n* = 11)	Male (*n* = 10)	Total (*n* = 21)
Age (years)	22 ± 1.6	23 ± 1.5	22.7 ± 1.5
BMI (kg/m^2^)	26 ± 5.3	24 ± 3.5	25.2 ± 4.5
Waist-to-hip ratio	0.75 ± 0.15	0.87 ± 0.14	0.80 ± 0.15*
Waist-to-height ratio	0.57 ± 0.13	0.51 ± 0.05	0.53 ± 0.10*
Fat mass (% body weight)	37.1 ± 7.9	22.2 ± 6.1	30.0 ± 10.3*
Muscle mass (% body weight)	34.6 ± 4.2	44.0 ± 3.5	39.1 ± 6.1*
Physical activity levels (METs*min*week)	357 ± 140	743 ± 1287	540 ± 891
Resting heart rate (beats*min)	81 ± 8	76 ± 9	78 ± 8
Systolic blood pressure (mmHg)	114 ± 9	119 ± 10	116 ± 10
Diastolic blood pressure (mmHg)	75 ± 7	74 ± 7	75 ± 7

Data is presented in mean ± SD

*Abbreviation list*: *BMI* Body mass index

^*^indicates statistical significant differences between female and male (p˂0.05)

### Phenotypical measurements and exercise prescription

**Fig. 1:**
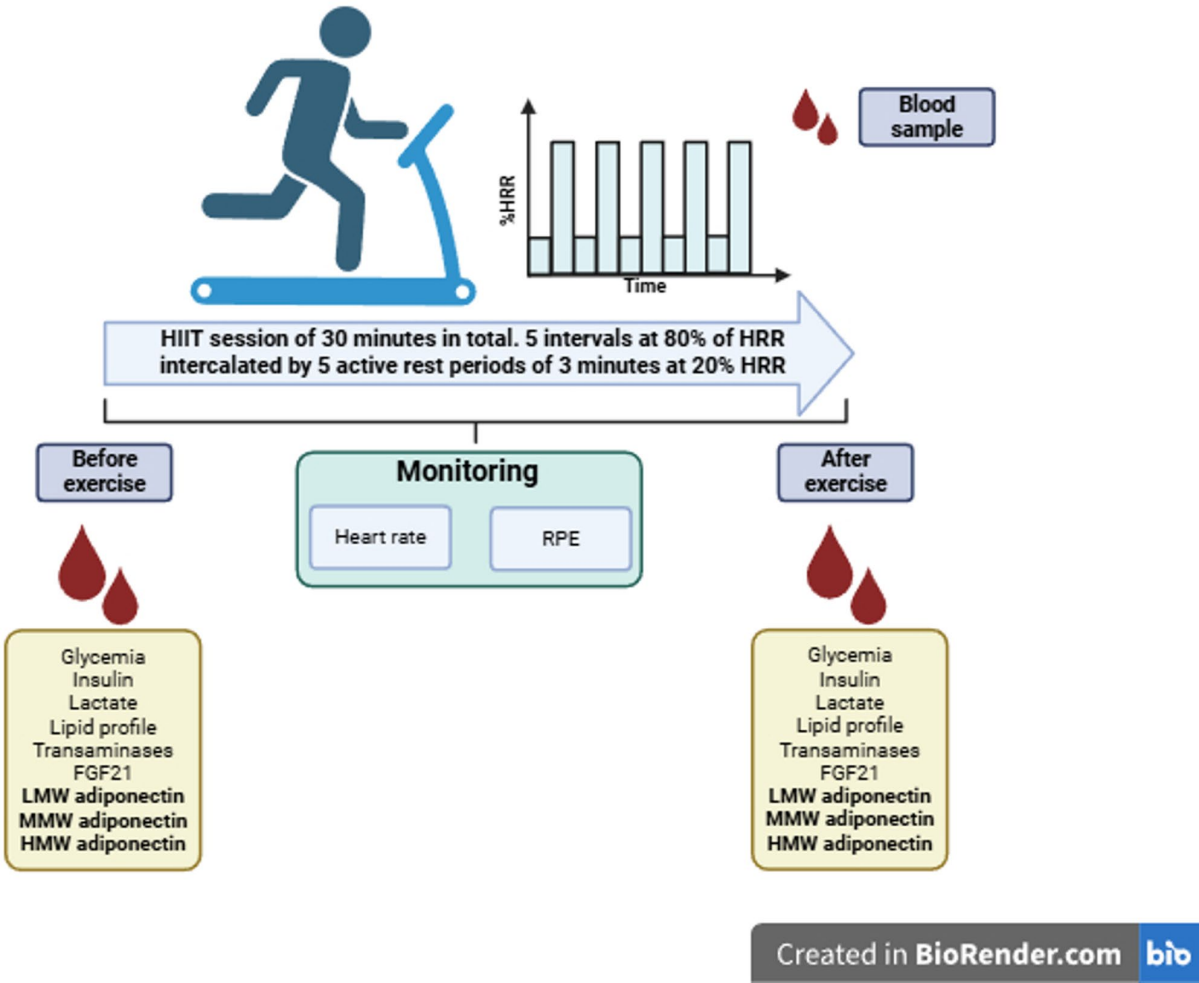
Study design. Abbreviation list: HRR: heart rate reserve; HIIT: high-intensity interval training; FGF21: fibroblast growth factor 21; LMW: low-molecular weight; MMW: medium-molecular weight; HMW: high-molecular weight; SmO2: muscle oximetry; RPE: ratings of perceived exertion. Figure created in Biorender.com

Prior to the exercise session, participants were instructed to maintain their usual dietary and hydration habits and to refrain from engaging in strenuous physical activity during the 72 h preceding the study day. Participants underwent anthropometric assessments, including weight, height, body mass index (BMI), waist circumference, and waist-to-hip and waist-to-height ratios. Body composition, specifically body fat and muscle mass relative to total body weight, was evaluated by bioelectrical impedance analysis (InBody^®^ 270). During the screening, physical activity levels during the week prior to the study were determined using the International Physical Activity Questionnaire (IPAQ) [31], previously validated for the local population [32]. The IPAQ captures an overall measure of physical activity by accounting for light, moderate, and vigorous activities across several activities, such as occupational, transportation-related, domestic, and leisure-time activities, which can overestimate activity, given that even light activities, such as walking, are considered. All sessions took place in a controlled environment (20–22 °C; ~60% humidity) between 15:00 and 17:00 h. Following five minutes of seated rest, physiological parameters were recorded. Heart rate (HR) was measured using a Polar^®^ H10 heart rate monitor, blood pressure (BP) was obtained using an OMRON^®^ sphygmomanometer, and oxygen saturation (SatO2) was assessed with a Nonin^®^ Onyx Vantage pulse oximeter. Perceived exertion was evaluated using the original Borg Rating of Perceived Exertion (RPE) scale [33]. Additionally, muscle oxygen saturation (SmO2) was continuously monitored through a wireless muscle oximeter (Moxy^®^ 5), positioned on the belly of the right vastus lateralis muscle. The HIIT protocol consisted of six 3-minute intervals at 80% of heart rate reserve (HRR), each followed by 3 min of active recovery involving slow walking, aiming to reduce heart rate toward approximately 20% HRR. Throughout the session, HR, SmO2, and RPE were continuously tracked and recorded every minute (Fig. 1). The speed and slope of the treadmill were adjusted individually before the session in order to achieve the target intensities. HRR was calculated with the Karvonen formula [34] and the 80% HRR was the target peak during each work bout, not at the start. All equipment was updated to its last firmware to secure its accuracy.

### Laboratory tests

Prior to the exercise session, blood lactate levels were assessed via finger prick using reactive strips and an AccuTrend Plus^®^ device. Venous blood samples were collected from the antecubital vein into two separate tubes: a 2-mL EDTA tube and a tube without anticoagulant containing separator gel. These samples were used for standard biochemical analyses including glucose, insulin, and calculation of the HOMA-IR index. All laboratory assessments were performed at the Clinical Laboratory of Universidad Austral de Chile following standardized procedures. Immediately after completion of the HIIT session (within 4 min post-exercise), additional venous blood samples and lactate measurements were obtained. This timing was selected due to the rapid post-exercise decline in blood lactate levels, which can decrease by approximately 50% within 4 min in healthy individuals [35].

### Adiponectin and western immunoblotting

As previously described [36], circulating adiponectin levels were measured using Western immunoblotting. Briefly, 0.5 µL of plasma was mixed with 2.5 µL of 6× loading buffer and 12 µL of RIPA buffer, and the mixture was loaded onto a polyacrylamide gradient gel (Bio-Rad^®^, catalog number 1704158). Protein separation was carried out by electrophoresis at 80–120 W for approximately 60 min. The separated proteins were then transferred onto a nitrocellulose membrane (Bio-Rad^®^, catalog number 1704158) using a semi-dry transfer system (Trans-Blot^®^ Turbo™, Bio-Rad^®^, USA).

Following transfer, membranes were blocked for one hour with 5% bovine serum albumin (BSA) in TBST buffer, washed with TBST, and incubated overnight at 4 °C with a rabbit monoclonal primary antibody against human adiponectin (1:1000 dilution; Cell Signaling^®^, catalog number 2789 S). The next day, membranes were washed again in TBST and incubated for one hour at room temperature with a peroxidase-conjugated secondary antibody (anti-rabbit IgG, 1:2500 dilution; Cell Signaling^®^, catalog number 7074 S). After a final wash, membranes were developed using a chemiluminescent substrate (Clarity™ Western ECL substrate, Bio-Rad^®^, catalog number 170–5061) and visualized using a densitometric imaging system (Clinx, Clinx Science Instruments Co^®^, China). Quantification of adiponectin bands corresponding to the LMW, MMW, and HMW isoforms was performed using ImageJ software. Total protein loading was verified by staining the entire membrane with Ponceau red, a method previously validated for this purpose [37]. Ratios between the adiponectin isoforms were calculated to potential isoform redistribution after exercise as previously described in the context of metabolic dysfunction [38, 39]. Adiponectin antibody was validated using positive control (isolated human adiponectin protein).

### Statistical analysis

Quantitative variables were expressed as means and standard deviations, whereas qualitative variables were presented as absolute frequencies. Data normality was assessed with the Kolmogorov-Smirnov test by sex. Comparisons between pre- and post-training measurements were performed using the Wilcoxon test. Statistical significance was set at a p-value less than 0.05 for all analyses. Data analyses were conducted using SPSS (version 20) and GraphPad Prism (version 8) software.

## Results

**Fig. 2:**
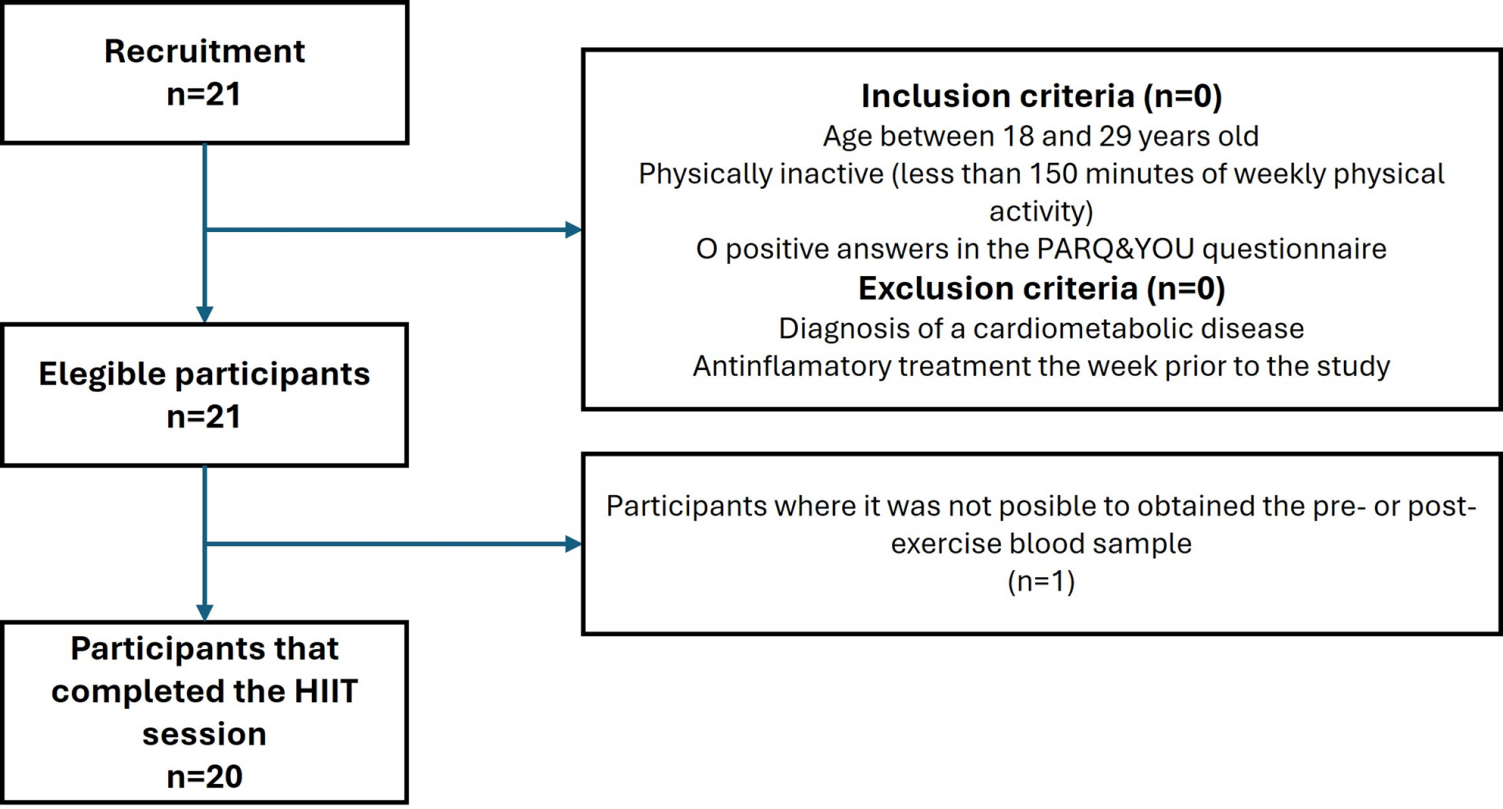
Study flowchart

A total of 21 participants (Fig. 2) completed the HIIT session without any adverse events. Of these, 11 were women, and the participants had a mean BMI of 25.2 kg/m². Body composition analysis showed that 30% of their body weight was fat mass, while 39% was muscle mass. Their self-reported physical activity levels averaged 540 METs·min/week, and both resting heart rate and blood pressure were within the normal range (Table 1).

**Fig. 3:**
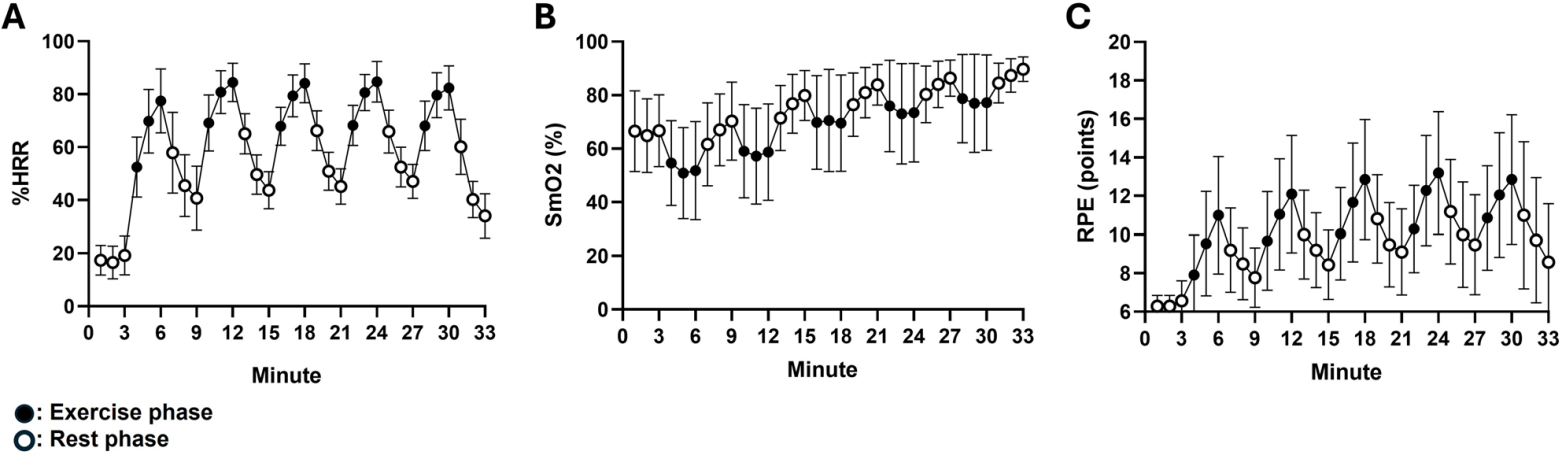
Cardiovascular responses to the high-intensity interval training session. Each point represents the mean and the bars the standard deviation of each minute of the exercise session. **A** percentage of heart rate reserve (%HRR) used (target intensity reached at the peak of each bout); **B** muscle oximetry (SmO2); **C** ratings of perceived exertion (RPE) by the original Borg’s scale. Abbreviation list: %HRR: percentage of the heart rate reserve used; SmO2: muscle oximetry; RPE: ratings of perceived exertion

During the HIIT session, participants achieved the target exercise intensity, particularly in terms of % heart rate reserve (%HRR). Additionally, quadriceps muscle oximetry displayed an intervallic pattern, aligned with subjective effort perception during the exercise intervals (Fig. 3). Regarding metabolic effects, significant post-exercise increases were observed in circulating No-HDL cholesterol, GPT, and lactate levels (both *p* < 0.05; Table 2), whereas insulin and VLDL cholesterol remained unchanged. To complement the interpretation of statistical significance, we examined the magnitude and confidence intervals of observed effects using Cohen’s dz for all metabolic variables. Post-exercise plasma lactate exhibited a very large effect size (dz = 1.164, 95% CI: 0.568 to 1.759), GPT levels increased with a moderate effect size (dz = 0.515, 95% CI: 0.016 to 1.015), and Non-HDL cholesterol also showed a moderate effect size (dz = 0.464, 95% CI: −0.03 to 0.958). We conducted exploratory sex × time interaction analyses via 2 × 2 ANOVA. However, no significant sex effects were observed (data not shown).

**Table 2:** Effect of exercise on plasma parameters

	Female (*n* = 11)		Male (*n* = 10)		All (*n* = 21)		
**Parameter**	**Pre**	**Post**	**Pre**	**Post**	**Pre**	**Post**	**Effect size (Cohen´s d [IC 95%])**
Glycemia (mg/dl)	92 ± 10	99 ± 33	94 ± 8	93 ± 10	93 ± 9	96 ± 24	0.156 [−0.315−0.627]
Insulin (µUI/ml)	47.5 ± 63.7	26.7 ± 28.1	20.7 ± 13.9	17.0 ± 15.8	34.1 ± 46.9	21.9 ± 22.7	−0.435 [−0.926−0.055]
Triglycerides (mg/dl)	130 ± 101	122 ± 76	143 ± 82	143 ± 83	137 ± 90	133 ± 79	−0.126 [−0.596−0.344]
Total cholesterol (mg/dl)	174 ± 30	164 ± 42	163 ± 25	167 ± 29	169 ± 28	166 ± 35	−0.106 [−0.576−0.363]
HDL (mg/dl)	54 ± 23	58 ± 23	48 ± 8	49 ± 9	51 ± 12	54 ± 18	0.204 [−0.269−0.677]
LDL (mg/dl)	98 ± 17	88 ± 29	87 ± 26	90 ± 30	93 ± 22	89 ± 29	−0.159 [−0.63−0.312]
VLDL (mg/dl)	23 ± 16	36 ± 35	29 ± 16	28 ± 16	26 ± 16	32 ± 27	0.238 [−0.237−0.713]
No-HDL cholesterol (mg/dl)	121 ± 22	125 ± 21	115 ± 28	119 ± 33	118 ± 25	122 ± 27*	0.464 [−0.03−0.958]
GOT (UI/L)	24 ± 7	22 ± 5	25 ± 11	28 ± 12	24 ± 9	25 ± 9	0.120 [−0.35−0.59]
GPT (UI/L)	20 ± 5	21 ± 5	38 ± 34	39 ± 34	29 ± 26	30 ± 25	0.515 [0.016–1.015]
Lactate (mmol/L)	2.1 ± 1.0	5.7 ± 2.1*	2.5 ± 1.8	6.1 ± 3.3*	2.3 ± 1.5	5.9 ± 2.7*	1.164 [0.568–1.759]

Values are presented in terms of mean ± SD

*Abbreviation list*: *HDL* High density lipoprotein, *No-HDL* Total cholesterol minus HDL, *LDL* Low density lipoprotein, *VLDL* Very-low density lipoprotein, *GOT* Glutamic-oxaloacetic transaminase, *GPT* Glutamate pyruvate transaminase

^*^p < 0.05 in comparisons pre vs. post exercise values

**Fig. 4:**
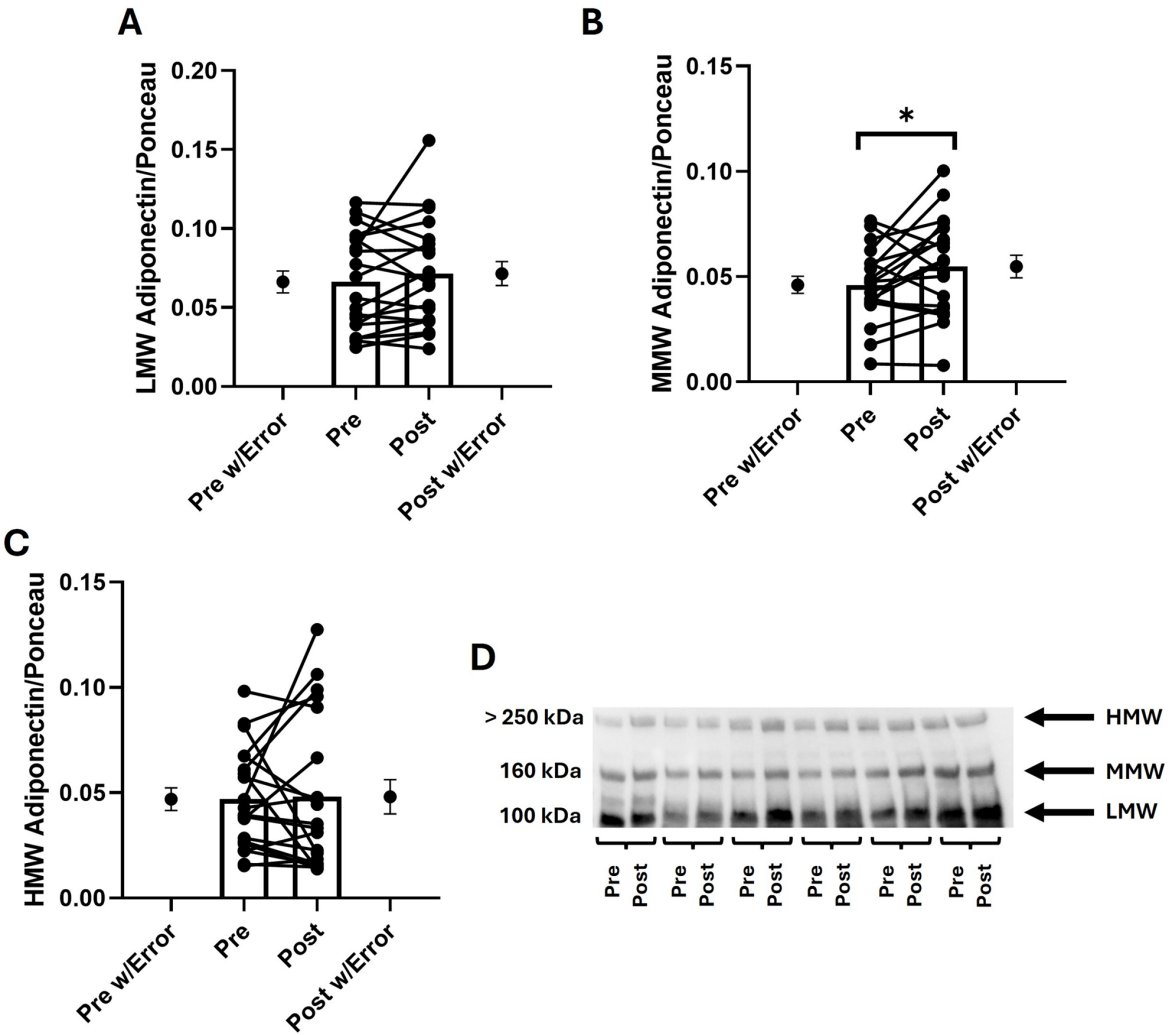
Effects of a HIIT session on adiponectin isoforms. **A** low-molecular weight (LMW) adiponectin; **B** medium-molecular weight (MMW) adiponectin; **C** high-molecular weight (HMW) adiponectin; **D** representative blots with the three adiponectin isoforms. All values are normalized by the total protein loading (Ponceau Red staining), each dot united by a line represents the pre- and post-exercise values of the same participant, while the dots with the error bars at the Pre-post w/Error indicate the mean and SEM of the whole group. ^*^: *p* < 0.05, indicate significant differences between the pre- and post-exercise values. Full-length blots/gels are presented in Supplementary File 1

**Fig. 5:**
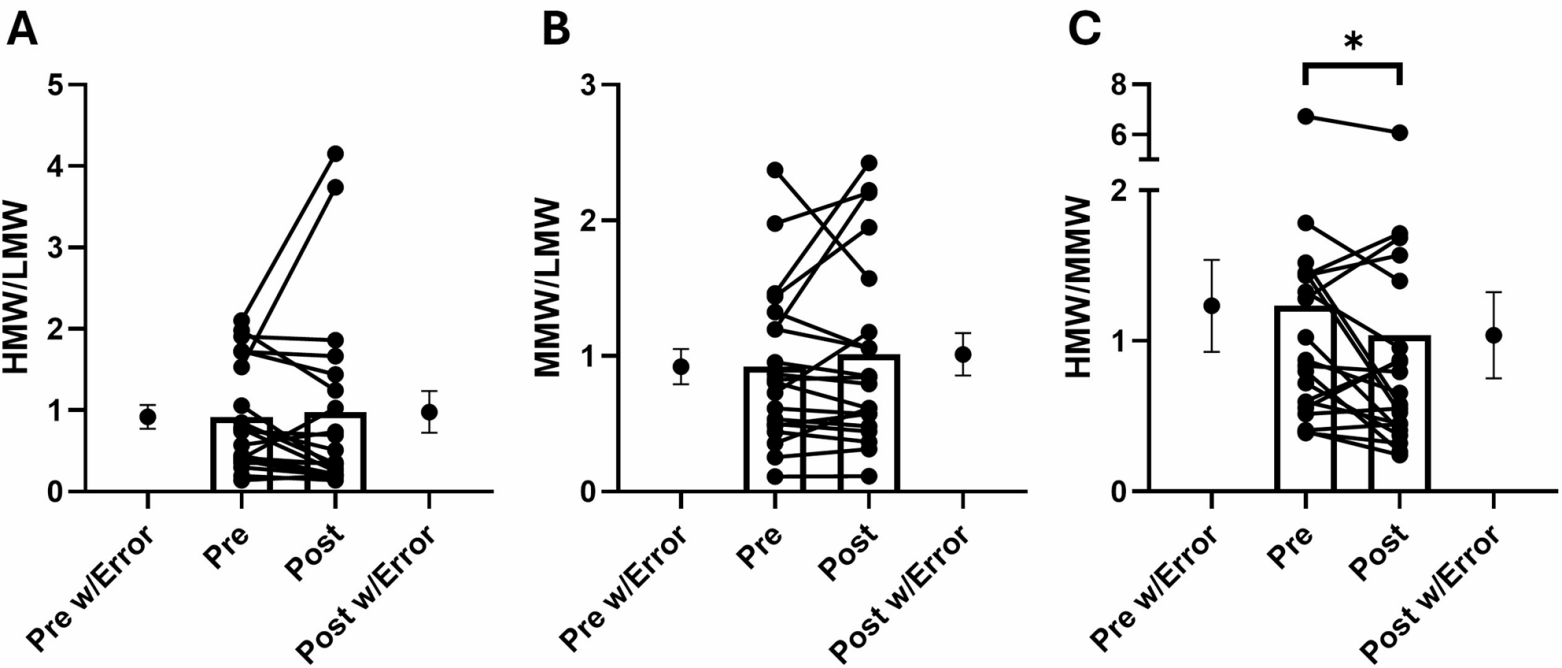
Effects of a HIIT session on the ratio of adiponectin isoforms. **A** ratio between high-molecular (HMW) and low-molecular weight (LMW) adiponectin; **B** ratio between medium-molecular (MMW) and LMW adiponectin; **C** ratio between HMW and MMW adiponectin. Each dot united by a line represent the pre- and post-exercise values of the same participant, while the dots with the error bars at the Pre-post w/Error indicate the mean and SEM of the whole group. ^*^: *p* < 0.05, indicate significant differences between the pre- and post-exercise values

Following the HIIT session, a significant increase was observed in MMW adiponectin (~ 19%; *p* < 0.05; dz = 0.49 [95% CI: −0.02 to 1.00]), whereas LMW (dz = 0.26 [95% CI: −0.22 to 0.73], *p* > 0.05) and HMW (dz = 0.04 [95% CI: −0.43 to 0.51], *p* > 0.05) isoforms remained unchanged (Fig. 4). Additionally, analysis of the isoform ratios revealed a significant decrease in the HMW/MMW ratio (−16%; dz = −0.49, 95% CI: −0.99 to 0.00, *p* < 0.05) after the exercise session. In contrast, the HMW/LMW (dz = 0.08, 95% CI: −0.39 to 0.55, *p* > 0.05) and MMW/LMW ratios (dz = 0.22, 95% CI: −0.26 to 0.69, *p* > 0.05) remained unchanged (Fig. 5). These data collectively indicate a selective and acute enhancement of the MMW isoform in response to HIIT, while the HMW isoform remained stable.

It is worth noting that after analyzing the data from male and female participants separately, we did not find any differences between the pre- and post-exercise values in any of the adiponectin isoforms (*p* > 0.05, data not shown), however, it is worth noting that sex-based subgroup analyses are exploratory and underpowered, thus interpreted cautiously. Moreover, outliers were assessed, and no data points were removed.

## Discussion

The present study aimed to evaluate the acute metabolic effects of a single HIIT session in insufficiently active young adults, with a particular focus on adiponectin isoforms and metabolic markers. Our findings showed a significant increase in MMW adiponectin (~ 19%; *p* < 0.05) following the HIIT session, while no changes were observed in LMW or HMW adiponectin levels. Additionally, we found a significant decrease in the HMW/MMW ratio (−16%; *p* < 0.05). Secondarily, we also observed increases in the plasma levels of No-HDL cholesterol post-exercise. It is worth noting that the HIIT protocol was individually adjusted in terms of treadmill speed and slope to achieve the prescribed intensity, as recommended in recently [40]. Moreover, the protocol was well tolerated, with no adverse events reported.

Adiponectin has been classified as an exerkine due to accumulating evidence demonstrating that exercise can elevate its circulating levels. As early as 2004, Kriketos et al. reported a substantial increase (260%) in total adiponectin following just two to three sessions of moderate-intensity exercise in overweight and obese males [41]. Subsequent studies from our group [42] and others [43, 44] have corroborated these findings in similar populations, indicating that these acute effects persist beyond the exercise session. For instance, plasma adiponectin remained elevated 90 min after a single HIIT session in overweight and obese young women [26]. However, the effects of exercise on specific adiponectin isoforms remain unclear. In older adults with insulin resistance, 12 weeks of supervised exercise significantly reduced MMW adiponectin without altering LMW or HMW isoforms [39]. These findings differ from our results, potentially due to age-related variations among participants. For example, exhaustive exercise in young adults has significantly elevated total and HMW adiponectin [25], a response not observed in middle-aged males following the same protocol. Similarly, middle-aged men with abdominal obesity showed a reduction in combined LMW and MMW adiponectin after a single high-intensity exercise session, but no change in HMW adiponectin [45]. Notably, the mean participant age in that study was 54 years. Additional factors, including metabolic status, exercise duration, and intensity, may also influence adiponectin isoform responses. Supporting this, our group’s randomized controlled trial demonstrated significant increases in LMW adiponectin following four weeks of high-intensity interval training among bariatric surgery candidates [42]. In contrast, one session of moderate-intensity continuous exercise in physically inactive young adults did not modify circulating levels of any adiponectin isoforms [27]. These observations suggest that exercise-induced adiponectin responses may depend on both duration and intensity. Future research should control and compare these variables closely, along with participant age, to provide clearer insights.

Finally, sex-specific differences in adiponectin regulation have been previously documented [46]. In human adipocytes it was seen that both male and female serum induced a down-regulation of adiponectin production, with a stronger effect seen after male serum treatment [47]. Such hormonal differences, together with variations in body fat distribution—subcutaneous predominance in women versus visceral in men, may contribute to distinct baseline adiponectin profiles and potentially to different exercise-induced responses. In our exploratory analysis, no significant sex × time interaction or within-sex differences were observed for any adiponectin isoform. This likely reflects the limited statistical power of our sample rather than the absence of physiological divergence. These hypotheses merit verification in larger, adequately powered studies that control for menstrual phase, hormonal contraceptive use, and body composition. Moreover, considering that acute exercise alters plasma volume, a limitation of this study is the absence of hemoglobin and hematocrit measurements to correct for potential hemoconcentration effects, which may differentially influence comparisons between sexes. Future studies should include these measures to accurately characterize sex-specific responses in adiponectin isoform dynamics.

The observed changes in adiponectin isoforms may be attributed to multiple physiological mechanisms, including acute muscle signaling triggered by exercise, adipose tissue responsiveness, and hormonal regulation. HIIT activates AMP-activated protein kinase (AMPK) and peroxisome proliferator-activated receptor gamma (PPARγ) pathways, both crucial regulators of adiponectin secretion from adipose tissue and skeletal muscle [18, 19, 48]. The increase in MMW adiponectin noted in our study indicates a favorable metabolic response, as this isoform is linked to improved insulin sensitivity and lipid metabolism [20], especially when compared to lower molecular weight isoforms (e.g., LMW adiponectin). The absence of changes in HMW adiponectin contrasts with findings from studies on trained athletes performing exhaustive exercise [25]. This discrepancy suggests that training status may significantly affect adiponectin isoform responses, as our study included insufficiently active participants. Moreover, increases in HMW adiponectin have been reported in individuals living with obesity following a ~ 10% reduction in body weight [49], suggesting that in the context of metabolic dysfunction, longer-term interventions may be necessary to elicit changes at this isoform level. Several biological factors influence adiponectin oligomerization, potentially explaining the isoform distribution observed here. The endoplasmic reticulum (ER) plays an essential role in the post-translational modifications (such as hydroxylation and glycosylation) necessary for forming higher molecular weight adiponectin isoforms [50]. Given that HIIT induces oxidative stress and transient hypoxia [51], these conditions may temporarily impair endoplasmic reticulum (ER) function, thereby affecting adiponectin oligomerization [52]. However, such brief acute stimuli may not be sufficient to promote the assembly of adiponectin into its HMW multimeric form—an aspect that warrants further investigation. Additionally, inflammatory responses in adipose tissue and cytokine activity—particularly involving tumor necrosis factor-alpha (TNF-α) and interleukin-6 (IL-6)—can modulate adiponectin secretion [53] and oligomerization [54], potentially shifting isoform distribution toward increased MMW adiponectin rather than the HMW form [52].

The analysis of adiponectin isoform ratios, such as HMW/MMW, was performed to explore potential shifts in the relative distribution of oligomeric forms following acute HIIT. Among these, we observed a significant decrease in the HMW/MMW ratio, suggesting a transient redistribution favoring medium molecular weight forms over high molecular weight adiponectin. While such shifts may reflect acute post-translational or secretion-related changes in adiponectin multimerization [55], they should be interpreted cautiously. In our study, the observed reduction in the HMW/MMW ratio primarily reflected the isolated increase in MMW adiponectin, with HMW remaining unchanged. Therefore, the biological meaning of ratio-based outcomes must be contextualized alongside absolute changes in each isoform. Furthermore, in studies with small sample sizes, the interpretation of ratio data is more susceptible to distortion by individual variability and outliers, which can exaggerate or mask subtle shifts. For these reasons, we report both the absolute values and the derived ratios, but recommend cautious interpretation and validation in larger, adequately powered samples.

As exploratory outcomes, given its association with adiponectin production, lipid profile and lactate [23, 24] were assessed along with the effect of one HIIT session on these parameters. The increase in lactate observed after the HIIT session was expected, as high-intensity exercise promotes the use of glucose and muscle-stored glycogen through anaerobic pathways, leading initially to the production of lactic acid and subsequently lactate [56]. Interestingly, these well-documented increases in lactate may be linked to the elevations in cholesterol observed in our participants. Early studies have shown that lactate can stimulate cholesterol biosynthesis in hepatocytes by activating acetyl-CoA carboxylase [57]. Additionally, similar lactate-induced upregulation of lipid synthesis has been reported in cancer cells [58] and in viruses [59], suggesting that this may be a conserved biological function of lactate. However, the clinical relevance of this mechanism in the context of exercise among individuals with obesity requires further investigation.

This study has several strengths. Notably, the exercise protocol and intensity were well-controlled among participants, changes in different adiponectin isoforms were reported, and metabolic parameters were directly measured. However, this study has several limitations. These include a small sample size, the lack of a longer post-exercise follow-up period, and the absence of dietary control or standardized fasting duration prior to the exercise session, which may have influenced glucose and lipid measurements. Additionally, the lack of a non-exercising control group limits the ability to attribute observed changes solely to the HIIT intervention. No corrections for multiple comparisons (e.g., by sex or adiposity level) were applied due to limited statistical power, which also represents a limitation given the known sex-related variability in adiponectin responses [46]. Also, the presence of outliers in our small sample could hinder the generalizability of these results to a broader population. These issues should be addressed in future studies to strengthen the generalizability and mechanistic understanding of the findings.

## Conclusions

In summary, this study provides preliminary evidence regarding the acute regulation of adiponectin isoforms following a single session of HIIT in insufficiently active young adults. A significant increase was observed in the MMW isoform of adiponectin, whereas the HMW and LMW isoforms remained unchanged. These findings suggest that MMW adiponectin could be a more responsive marker to acute exercise stimuli than other isoforms, although this interpretation should be viewed cautiously given the exploratory nature of the study. In addition, concurrent changes in lactate and lipid parameters indicate short-term metabolic effects of HIIT in this population. However, due to the small sample size, absence of a control group, and lack of dietary standardization, the results should be considered hypothesis-generating rather than conclusive. Future randomized and adequately powered studies are warranted to confirm these observations and to clarify the temporal dynamics and metabolic significance of isoform-specific adiponectin responses to exercise.

## Supplementary Information

Supplementary Material 1

## Data Availability

Data are available upon request to the corresponding author at sergio.martinez@uss.cl.

## Abbreviations

HIIT: High-intensity interval training
HRR: Heart rate reserve
LMW: Low-molecular weight
MMW: Medium-molecular weight
HMW: High-molecular weight
HDL: High-density lipoprotein
LDL: Low-density lipoprotein
VLDL: Very-low-density lipoprotein
HOMA-IR: Homeostatic model assessment for insulin resistance
BMI: Body mass index
IPAQ: International physical activity questionnaire
SatO2: Oxygen saturation
RPE: Ratings of perceived exertion
SmO2: Muscle oxygen saturation
TBST: Tris-buffered saline with Tween 20
BSA: Bovine serum albumin
ER: Endoplasmic reticulum
TNF-α: Tumor necrosis factor alpha
IL-6: Interleukin 6
AMPK: AMP-activated protein kinase
PPARγ: Peroxisome proliferator-activated receptor gamma
GOT: Glutamic-oxaloacetic transaminase
GPT: Glutamate pyruvate transaminase
MET: Metabolic equivalent of task
