# Supplementary figures and images for: Acute effects of high-intensity interval training on adiponectin isoforms in inactive young adults: a quasi-experimental study
Source: BMC Sports Sci Med Rehabil. 2025 Dec 30;17:387. doi: 10.1186/s13102-025-01433-7 (PMC12755020; doi:10.1186/s13102-025-01433-7)

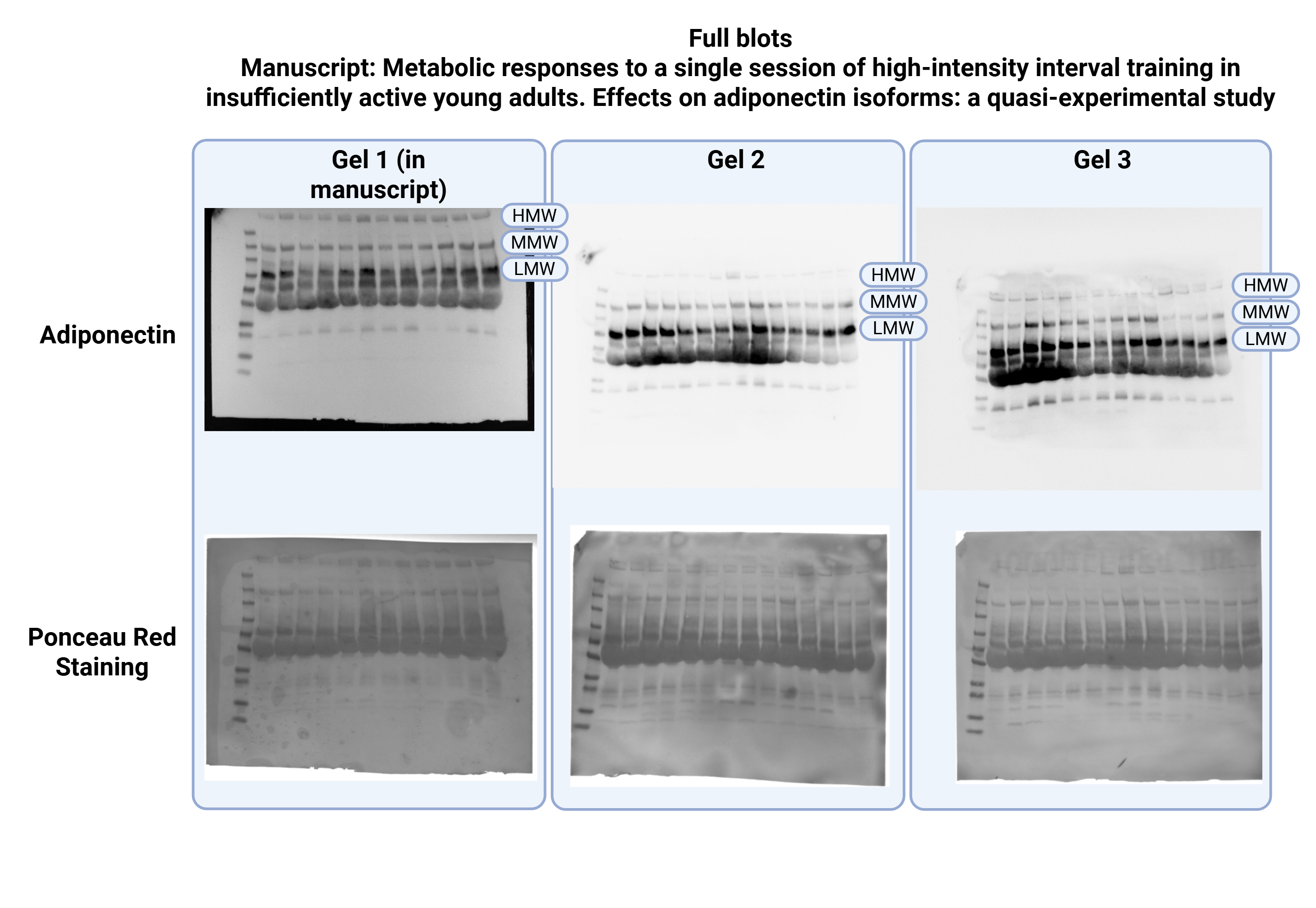

Supplement: Supplementary file 1 — Supplementary Material 1 [file 13102_2025_1433_MOESM1_ESM.png]
